# Methodology of assessment and reporting of safety in anti-malarial treatment efficacy studies of uncomplicated falciparum malaria in pregnancy: a systematic literature review

**DOI:** 10.1186/s12936-017-2136-x

**Published:** 2017-12-18

**Authors:** Makoto Saito, Mary Ellen Gilder, François Nosten, Philippe J. Guérin, Rose McGready

**Affiliations:** 1 WorldWide Antimalarial Resistance Network (WWARN), Oxford, UK; 2 Centre for Tropical Medicine and Global Health, Nuffield Department of Medicine, University of Oxford, Old Road Campus, Roosevelt Drive, Oxford, OX3 7FZ UK; 3 Shoklo Malaria Research Unit (SMRU), Mahidol-Oxford Tropical Medicine Research Unit, Faculty of Tropical Medicine, Mahidol University, Mae Sot, Tak, Thailand

**Keywords:** Malaria, Pregnancy, Safety, Methodology, Review

## Abstract

**Background:**

Considering the uncertainty of safety of anti-malarial drugs in pregnancy, efficacy studies are one of the few sources of clinical safety data. Complete safety evaluation is not usually incorporated in efficacy studies due to financial and human resource constraints. This review reports the methods used for the assessment of safety of artemisinin-based and quinine-based treatments in efficacy studies in pregnancy.

**Methods:**

Methodology of assessment and reporting of safety in efficacy studies of artemisinin-based and quinine-based treatment in pregnancy was reviewed using seven databases and two clinical trial registries. The protocol was registered to PROSPERO (CRD42017054808).

**Results:**

Of 48 eligible efficacy studies the method of estimation of gestational age was reported in only 32 studies (67%, 32/48) and ultrasound was used in 18 studies (38%, 18/48). Seventeen studies (35%, 17/48) reported parity, 9 (19%, 9/48) reported gravidity and 13 (27%, 13/48) reported both. Thirty-eight studies (79%, 38/48) followed participants through to pregnancy outcome. Fetal loss was assessed in 34 studies (89%, 34/38), but the definition of miscarriage and stillbirth were defined only in 11 (32%, 11/34) and 7 (21%, 7/34) studies, respectively. Preterm birth was assessed in 26 studies (68%, 26/38) but was defined in 16 studies (62%, 16/26). Newborn weight was assessed in 30 studies (79%, 30/38) and length in 10 studies (26%, 10/38). Assessment of birth weight took gestational age into account in four studies (13%, 4/30). Congenital abnormalities were reported in 32 studies (84%, 32/38). Other common risk factors for adverse pregnancy outcomes were not well-reported.

**Conclusion:**

Incomplete reporting and varied methodological assessment of pregnancy outcomes in anti-malarial drug efficacy studies limits comparison across studies. A standard list of minimal necessary parameters to assess and report the safety component of efficacy studies of anti-malarials in pregnancy is proposed.

**Electronic supplementary material:**

The online version of this article (10.1186/s12936-017-2136-x) contains supplementary material, which is available to authorized users.

## Background

Malaria in pregnancy affects both mother and fetus, regardless of whether the infection is clinically symptomatic or not [1–3]. Malaria in pregnancy can lead to higher risks of miscarriage, stillbirth, preterm birth (PTB), low birth weight (LBW), small for gestational age (SGA), and maternal anaemia and mortality [1–3]. PTB, LBW and SGA lead to a higher risk of perinatal mortality [4, 5]. Maternal mortality attributable to malaria is known to be higher in low transmission settings [2] because of the lower immunity against parasites, and may be increased as mid to high endemic areas reduce endemicity to low levels [6]. In the context of declining *Plasmodium falciparum* malaria prevalence and the emergence of resistant parasite strains of both *P. falciparum* and *Plasmodium vivax*, there is a great need for clarity on both efficacy and safety of anti-malarial drugs for the mother and fetus.

Safety reporting of new anti-malarials in pregnancy has not kept pace with release of new drugs in the face of resistance for the non-pregnant populations. The artemisinins are a particular case in point, as rodent and monkey studies consistently reported fetal resorption (embryotoxicity) and congenital abnormalities (teratogenicity). Fetal resorption by artemisinins was considered to be mediated by the depletion of embryonic erythroblasts [7]. For fetal resorption, an added concern was that the level at which adverse effects were seen was very close to the therapeutic range used in humans [7]. The ‘no observed’ adverse effect level for artemisinin was at 4 mg/kg/day in monkeys although these adverse events were reported at much longer gestational exposure times than typically given for treatment of malaria (e.g. 12–30 gestational days in monkey studies versus 3–7 days for malaria treatment) [7–12]. Similarly, reticulocytes were reported to be decreased in humans at therapeutic doses, even though human reticulocytes were shown to be less sensitive to artemisinin than embryonic erythroblasts in animals [7]. Major congenital abnormalities were observed in the cynomolgus monkey studies including skeletal (e.g. shortening of the long bones) and cardiovascular malformations [11]. These concerning findings in animal studies emphasize the importance of assessment and reporting of fetal loss, infant cardiac assessment, and infant length following treatment in humans.

Safety of anti-malarials in non-pregnant participants suggested by the World Health Organization (WHO) or the Clinical Data Interchange Standards Consortium (CDISC) includes assessment of symptoms, complete blood count, blood biochemistry, urinalysis, electrocardiogram (ECG) and serious adverse events (deaths, hospitalisations and disability) [13, 14]. In addition to these general assessments, specific safety outcomes such as miscarriage, stillbirth, congenital abnormality, PTB and SGA are important following anti-malarial treatment in pregnancy. The safety of anti-malarials during human pregnancy has been assessed across continents and in different types of clinical studies [12, 15–21].

The prospective nature of efficacy studies provides a framework for capturing safety data, although the detailed assessment and reporting of safety may not be the main interest. Pathological confirmation of the infection parameters, particularly the parasitological confirmation of malaria parasites, provides an advantage in that characteristics of the disease can be considered in addition to drug treatment. While animal studies raised important concerns, they may have overestimated the risk for humans [21–23] so information from every available source should be utilized to clarify clinical safety of anti-malarial drugs in pregnancy.

This manuscript reviewed the methodology of safety assessment and reporting in the context of artemisinin-based (ABT) and quinine-based anti-malarial treatment (QBT) efficacy studies in pregnancy, with a view to developing a guideline for systematic use in future pregnancy studies.

## Methods

A systematic literature review following the methodology described in the PRISMA statement [24] was conducted to identify studies measuring the efficacy of ABT and QBT in pregnant women with parasitologically confirmed uncomplicated falciparum malaria, regardless of trimester or clinical symptoms. Seven different search databases (MEDLINE, Embase, Global Health, Cochrane Library, Scopus, Web of Science and LILACS) and two clinical trial registries (ICTRP and ClinicalTrial.gov) were used. This review was registered to PROSPERO (CRD42017054808) and the search terms and conditions are available in Additional file 1.

Briefly, five elements were used: malaria; pregnancy; treatment (ABT or QBT); study design (interventional or observational cohort); and efficacy. No limitation was set for publication year and language. The search was conducted between July 2016 and January 2017. Two independent assessors checked eligibility and any discrepancy was resolved by a second assessment.

After screening, the following data were extracted: demographic information of study (year, country, study design, study drugs and eligibility criteria), reporting of outcome assessment and definition (pregnancy outcomes and other safety outcomes), reporting of risk factors for the pregnancy outcomes, and the methodology of assessment of variables (method of estimating gestational age and anthropometric measurements). Maternal death, laboratory changes including maternal anaemia, fetal loss, preterm birth, anthropometric measurements, congenital abnormality and neonatal death were reviewed.

Uncomplicated malaria was defined as malaria infection without features of severe malaria [25]. Trimester of the pregnancy was defined as the first (< 13 completed weeks), the second (14 weeks–27 completed weeks) and the third (from 28 weeks until delivery). Definitions for miscarriage (spontaneous abortion), stillbirth, preterm delivery, low birth weight, neonatal death were summarised across the studies.

## Results

A total of 48 studies assessing treatment efficacy of ABT or QBT for uncomplicated falciparum malaria in pregnancy were retrieved (see Additional file 2). A total of 7111 women with confirmed falciparum malaria were enrolled in those trials, 6147 and 964 participants were treated with ABT or QBT, respectively. Forty-one were published, five presented at conferences and two registered but not yet published.

There were 22 randomized control trials (RCTs) comparing two or more treatment regimens [26–48], ten pharmacokinetic (PK) studies including clinical outcome assessment [49–59], six single arm interventional studies [60–65] and ten observational cohort studies [66–76] (see Additional file 3).

### Obstetric care and characteristics of participants

#### Gestational age

Thirty-two studies (67%, 32/48) reported estimated gestational at the time of the malaria episode in weeks (n = 31) or in months (n = 1) [37]. Nine studies (19%, 9/48) did not report gestational age but reported the trimester of the malaria episode [26, 27, 38, 40, 41, 54, 59, 66, 75]. One RCT [29] did not report gestational age or trimester, and information was not available from six unpublished studies [44, 47, 48, 55, 64, 72].

Thirty-two studies (67%, 32/48) reported the method for estimating gestational age (see Additional file 3). Among them, ultrasound was used for at least some women in 18 studies (38%, 18/48). Newborn examination for gestational age estimation was used in another 12 studies (25%, 12/48) using the Dubowitz (19%, 9/48) or Ballard score (6%, 3/48). Last menstrual period and symphysis fundal height were the only methods of estimating gestational age in two studies (4%, 2/48). Quality control of gestational age estimation was mentioned in two studies [41, 53].

#### Parity and gravidity

Seventeen studies (35%, 17/48) reported parity, 9 (19%, 9/48) reported gravidity and 13 (27%, 13/48) reported both (see Additional file 3). Only three published studies (6%, 3/48) did not report either. Information was not available from six unpublished studies.

### Antenatal follow-up after malaria efficacy assessment

Thirty-eight studies (79%, 38/48) followed participants until pregnancy outcome (see Additional file 3). Ten studies did not specify the follow-up of participants after the primary endpoint of efficacy (e.g. day 28–63).

### Assessment of maternal safety

#### Maternal deaths

Maternal deaths were reported in 13 out of 46 published or presented studies, including four studies with no maternal deaths (28%, 13/46). [26, 31, 33, 36, 37, 39–41, 43, 61, 63, 68, 74].

#### Maternal anaemia and other laboratory investigations

Thirty-nine (81%, 39/48) studies reported haematological assessment at least once during the study. Of them, 20 studies (51%, 20/39) used haematocrit [28–33, 36, 48, 49, 51–53, 66–71, 73], 17 studies (44%, 17/39) used haemoglobin [34, 35, 37, 39–41, 43, 50, 54, 56, 57, 60–64, 75] and two studies (5%, 2/39) used both [46, 65]. Four published studies did not report haematological assessment [26, 38, 59, 74] and information was not available in five unpublished studies [27, 44, 47, 55, 72]. Twenty-five studies (52%, 25/48) continued assessing maternal anaemia after the primary endpoint of efficacy (e.g. day 28–63) regularly or only at delivery [30–33, 35, 36, 39, 41, 43, 48, 49, 52–54, 60, 61, 63, 64, 66–68, 70, 71, 73, 75]. Seventeen (35%, 17/48) studies specified the use of haematinics: 15 provided iron and folic acid [31, 33, 35–37, 39, 41, 48, 52, 53, 67, 68, 70, 73, 75]; and two did not explain the details [38, 54].

Reporting of other laboratory investigations was summarized in Additional file 4.

### Assessment of fetal safety

#### Fetal loss

Fetal loss was assessed in 34 studies (89%, 34/38) out of the 38 which followed through to pregnancy outcome (see Additional file 5) and not reported in three published studies [54, 62, 69] and one registered trial [47]. An additional two studies (20%, 2/10) out of ten studies without specified follow-up past the efficacy endpoint still reported pregnancy loss during the study period [28, 56].

Miscarriage was defined in 11 studies (32%, 11/34). Miscarriage was defined as fetal loss before 28 weeks’ gestation in ten studies [33, 35, 43, 50, 60, 61, 66–68, 70]. One study defined miscarriage as fetal loss < 20 weeks and intrauterine fetal death as fetal loss > 20 weeks [39]. Six studies did not define the term but reported miscarriage [26, 31, 36, 41, 45, 74].

Stillbirth was defined in seven studies (21%, 7/34) [33, 35, 39, 43, 66, 68, 70]. Six studies used the cut-off of 28 weeks gestation or more, and one study used 21 weeks [39]. Nine studies did not give a definition but reported stillbirth [32, 36, 37, 41, 44, 45, 66, 74, 75].

#### Preterm birth

Eleven studies reported the mean or median estimated gestational age at delivery [30–33, 35, 36, 39, 49, 52, 64, 66–68, 70, 71, 75] (see Additional file 5). Five studies only included singletons for the summary calculation [31, 33, 36, 68, 70]. Twenty-six studies (68%, 26/38) assessed PTB or prematurity of the newborn. Ten studies (26%, 10/38) did not report whether they assessed PTB or not [30, 37, 45, 51, 53, 54, 62, 66, 67, 69], and information was not available from two unpublished studies [44, 47].

PTB was defined in 16 studies (62%, 16/26). All of them used the cut-off of < 37 weeks’ gestation. Two used > 28 weeks as the lower boundary [50, 60] and one study differentiated those born < 28 weeks as severe prematurity [39]. Six of them defined PTB but did not report the results [31, 32, 35, 60, 68, 70]. Seven studies did not define but did reported PTB [26, 27, 52, 61, 64, 71, 74]. In two studies, all deliveries were full-term [29, 63]. One unpublished study plans to assess PTB [48].

#### Anthropometric assessment of newborns

Birth weight was assessed in 30 studies (79%, 30/38) (see Additional file 6). LBW was reported in 19 studies (63%, 19/30). LBW was defined in 18 studies (60%, 18/30) but one of them did not report the result [66]. Birthweight of less than 2500 g was used as the cut-off regardless of the estimated gestational age at delivery with an exception of one study which included 2500 g as LBW [51]. Two studies did not define LBW but reported the proportion [31, 32]. Four studies reported birth weight related to gestational age at delivery (i.e. small for gestational age comparing to the WHO growth curve [75] or body weight of term babies only [36, 39, 74]). Only singleton births were included in the calculation of the average birthweight in eight studies [31–33, 36, 41, 68, 70, 73], and women with multiple gestations were excluded from the study in four studies [35, 37, 43, 44]. All women delivered singletons in four additional studies [49, 51, 52, 71]. Gender of the newborns, which affects birth weight, was only reported to be assessed in six studies (16%, 6/38) [33, 48–50, 52, 75] and was used for body weight assessment in one of them [75].

Length of newborns was assessed in ten studies (26%, 10/38) and four of them (11%, 4/38) reported the results [33, 36, 64, 74]. Head circumference of newborns was assessed in 11 studies (29%, 11/38) and five of them (14%, 5/37) reported the results [33, 36, 61, 63, 74]. Length and head circumference were reported in term babies only in one study [74], and estimated gestational age was not considered in other studies.

The timing of anthropometric assessment was specified in 12 studies (32%, 12/38): within 24 h (n = 5) [30, 33, 35, 61, 63], 48 h (n = 1) [64], 72 h (n = 4) [36, 41, 52, 73] and 5 days (n = 1) [49]. One registered trial specified the timing as ‘as soon as possible after delivery’ [48]. Five studies (17%, 5/30) specified the minimal precision of the body weight scales used [31, 32, 52–54] and one study specified the minimum digit of the measurement [35]. Only 2 of 19 RCTs which followed women until delivery (10%, 2/19) stated that the physical assessment of babies was done by blinded investigators [36, 39].

#### Congenital abnormality

Thirty-two studies (84%, 32/38) reported the presence of any congenital abnormalities. Six studies (16%, 6/38) did not report whether there were any newborns with congenital abnormality [26, 34, 45, 62, 69, 75]. No studies reported that cardiac auscultation was specifically performed except one study in which newborn heart sounds were systematically documented [36]. Only one infant with congenital heart diseases was reported, and the estimated gestational age at drug exposure was 19 weeks [39]. Twenty-five studies (66%, 25/38) followed the newborns for a period (from 6 weeks to 3 years) after delivery to assess mortality, congenital abnormality and development (see Additional file 6). At least six different methods of developmental assessment [77–82] were used in eight studies [29, 30, 33, 36, 39, 48, 52, 74] and three other studies reported using developmental milestones [31, 32, 50].

#### Neonatal mortality and condition of newborns at birth

APGAR score was assessed in four studies (11%, 4/38) [30, 39, 41, 74], and only one of them reported the results [30]. Neonatal jaundice was assessed in seven studies (18%, 7/38) [29, 30, 35, 37, 41, 43, 74], and four of them reported the results [29, 30, 35, 37]. Neonatal death was defined in six studies (16%, 6/38) (see Additional file 5). Two of them reported early neonatal death defined by death within the first week of life [39, 74]. Four studies used a month: a month [53, 73], 27 days [35] and 32 days [36]. Perinatal death was defined in two studies (5%, 2/38). One study included miscarriage, stillbirth and neonatal death (within 27 days after birth) [35]. The other study defined it as death from 28 weeks gestational age until 1 week after delivery [60]. Sixteen studies (42%, 16/38) did not define but reported the deaths of newborns after delivery [26, 31–34, 37, 43, 44, 52, 60, 61, 63, 64, 66, 71, 75].

### Associated obstetric risk factors for adverse pregnancy outcomes

Risk factors for adverse pregnancy outcomes were not well-reported (see Additional file 7). The previous history of pregnancy loss was considered in two studies (4%, 2/48), which excluded women with a history of multiple miscarriage or stillbirths [37, 41]. All other studies (96%, 46/48) did not report the previous history of pregnancy loss. Except one study reporting the history of previous preterm birth to explain a neonatal death [33], the histories of previous preterm birth or low birth weight were not reported in any other studies. In 21 studies (44%, 21/48), women with known chronic diseases (including renal/hepatic/cardiac/mental diseases) were excluded from the study. One study reported the proportion of women with diabetes [74] and another study reported there were no women with chronic diseases included [56]. No studies reported the history of non-malarial febrile illness during the pregnancy but two studies excluded women with other diseases associated with fever [46] or severe underlying diseases presenting with fever [65]. Ten studies mentioned HIV status of the participants. Four studies included women with HIV [35, 37, 39, 45]. Two studies included women with HIV only if cotrimoxazole prophylaxis (and antiretroviral treatment) was not administered [41, 75]. Four studies excluded women with HIV [43, 44, 51, 57].

Smoking status was assessed in six studies (13%, 6/48) [36, 39, 48, 52, 53, 56]. One published study assessed the smoking status, but did not report it [39]. Women with a history of alcohol (or narcotic abuse) were excluded in four studies (8%, 4/48) [33, 36, 48, 49], and alcohol consumption was not reported in any other studies. One study reported the use of traditional medicine in the description of a stillbirth [35], and two studies excluded women who used herbal medicine in the past 4 weeks at enrolment [46, 65]. Marital status was reported in three studies (6%, 3/48) [35, 54, 75]. Consanguinity was reported as a possible reason for a stillbirth in a study [36]. Education level was reported in eight studies (17%, 8/48) [35, 37, 38, 43, 48, 51, 54, 75]. Maternal height was assessed in eleven studies (23%, 11/48) [30, 43, 46, 48, 50, 51, 53, 54, 56, 65, 67], and five of them also reported body mass index (BMI) [46, 51, 54, 56, 65]. Three studies reported BMI without the maternal height [36, 52, 57]. No studies reported any other nutritional status assessment. The number of antenatal care visits was not reported except one study reporting the proportion of women who attended the antenatal clinic at least two times [54].

## Discussion

This literature search revealed the variability of anti-malarial safety reporting in treatment efficacy studies in pregnancy. Understandably, pregnancy outcomes remain secondary to the primary objective of anti-malarial efficacy studies and reporting of safety outcomes may have been omitted or truncated to meet journal length restrictions. Safety assessment in the context of malaria is further stymied by the paucity of reliable background data on pregnancy outcomes and early childhood norms in the target populations in low and middle-income countries [15]. Methodological difficulties, variability of definitions and measurement of key indicators, such as gestational age and birth weight [83], and lack of integrated malaria, antenatal and delivery activities prevents confident pooling of published data which could potentially fill the gaps of current knowledge about the safety of anti-malarials in pregnancy.

Pregnancy outcomes can be affected by many factors. Malaria can affect pregnancy outcomes by acute disease effects (e.g. fever), acute and chronic effects (e.g. placental sequestration), and drug effects. Fever by non-malaria causes, common in tropical areas, can cause pregnancy loss or preterm labour [84]. Placental sequestration can cause inflammation of the placenta and reduction in the blood flow to the placenta, which lead to impaired placental function and intrauterine growth restriction [85–87].

For meaningful interpretation of pregnancy outcomes, gestational age needs specific attention [88] as it is necessary for interpretation of laboratory results, infant birth weight (as appropriate, large, or small for gestational age), as well as to evaluate the likelihood that a certain drug exposure could have a causative relationship with an adverse outcome. Preterm delivery and miscarriage are important outcomes associated both with infection and certain medications, and can only be evaluated with accurate estimation of gestational age. However, gestational age was assessed differently across the included studies and with each method there is invariably a degree of increasing error as pregnancy progresses: after 24 weeks ultrasound cannot reliably determine gestational age and last menstrual period becomes more difficult to recall. Gestational age assessment relies on quality control, which was often not reported. Modifying superficial and neurological items in clinical newborn gestational age assessment reduces the accuracy of these tests [89].

Laboratory results differ from non-pregnant participants due to the physiological changes of gestation [90]. Although haematologic markers were commonly assessed, the physiological change of haemoglobin over pregnancy [91] was not considered. A simple comparison between one measurement at the time of malaria and one at delivery may not estimate the effect of malaria or anti-malarials correctly. The impact of repeated malaria infection needs to be assessed.

As exposures to artemisinin derivatives in early pregnancy were shown to be related to cardiovascular abnormalities in animal studies [12], echocardiography should be ideally used but is rarely feasible in low resource settings. In the absence of echocardiography, serial cardiac auscultation and clinical exams could be used to identify murmurs, signs of congestive heart failure, or cyanosis. Unfortunately, affected children are often difficult to identify against the high background incidence of respiratory infections [92, 93]. Shortening of long bones is also reported in animal studies, and length of the newborn should be measured and compared to a standard that accounts for sex and an adequately estimated gestational age. Ongoing follow up and clinical exams to assess the development of infants can be useful to detect musculoskeletal abnormalities. Mode of feeding and other morbidities including malaria, need to be considered in the developmental assessment. In addition to the technical difficulties of developmental assessment, efficacy studies are unlikely to provide sufficient numbers of participants to assess for congenital abnormalities (which are rare) [94], and it will be necessary to combine data from these studies with information from other sources.

It is difficult to compare birthweight between studies and even between different study arms in anti-malarial efficacy trials. Assessment and comparison require a precise scale as the reported magnitude of reduction in birthweight is 35–310 g [1]. However, comparison with this precision is difficult because of the variability of methodology, inaccuracy of measurements and lack of information on confounding factors [83]. Firstly, birthweight for comparison should be measured in undressed live singleton newborns without any apparent congenital abnormalities within 24 h. Body weight on day 3–5 can physiologically be ≥ 10% lower than the birthweight [95, 96]. Accuracy and precision of the body weight scale also need to be specified before concluding the difference in different treatment arms. Secondly, birthweight is affected by the gestational age and sex. Preterm babies are more likely to be categorized as being LBW, but LBW does not necessarily equate to SGA [97]. LBW infants include a mixture of preterm and intra-uterine growth restricted babies, and these categories should be distinguished as they reflect different aetiologies and outcomes. The impact of malaria on birthweight is more accurately assessed using SGA [97]. International standards to assess birthweight adjusted for gestational age and sex are available such as INTERGROWTH-21st, which also covers populations in malaria endemic areas [88].

It is necessary to determine whether adverse outcomes are related to treatment rather than malaria. This can be only done when the risk is compared between different treatments or to a well-established population prevalence. Several risk factors for adverse pregnancy outcomes, such as smoking, hypertension, low BMI and primigravida were also reported in malaria endemic settings [97] and there are some other potential local risk factors such as traditional medicine and consanguinity. These should be recorded and assessed, especially in non-comparative studies [23]. Parity and gravidity were not consistently reported and if some manuscripts report parity and others gravidity, pooling of data becomes difficult. A distinction between antepartum stillbirth and intrapartum stillbirth is important, as intrapartum stillbirth is mainly affected by the availability and the level of delivery support [98]. Finally, the impact of the timing and frequency of malaria episodes and drug administration in each pregnancy need to be assessed with parasitological confirmation [99], and this is most appropriately done in prospective studies. A simple checklist of for reporting pregnancy-related information in anti-malarial efficacy studies is provided (Table 1).

**Table 1:** Recommendations for reporting anti-malarial drug safety in pregnancy in efficacy studies

Report the following:
Participants’ background
History of multiple miscarriages or stillbirths
History of preterm birth (< 37 weeks)
History of low birth weight (< 2500 g)
Chronic comorbidity (such as hypertension, diabetes mellitus, HIV and tuberculosis) depending on the prevalence in the area
Suggested risk factors include smoking status, alcohol consumption, other local social drugs, history of non-malaria febrile illnesses, concomitant medications, consanguinity, nutritional status and socioeconomic status (marital status and educational status)
Haematology
Haematinics (iron, folate) use: dose, date
Haematological measurements: haemoglobin/haematocrit and date
Fetal loss
Define miscarriage and stillbirth
Differentiate intrapartum and antepartum stillbirth
Preterm birth
Preterm birth should be clearly defined using standard definitions (preferably WHO)
Anthropometric assessment of infant
Use standard methods for measurement of birthweight, birth length, head circumference and for quality control of these measurements
Describe minimal precision of body weight/length scale
Control anthropometric outcomes by gestational age and sex. International standard of anthropometric outcomes is available (e.g. INTERGROWTH-21st)
Include normal singleton live births in summary anthropometric reporting
Separate reporting and analysis of multiple pregnancy, e.g. twins
Record the time interval between birth and anthropometric measurement
Congenital abnormality
Report details of congenital abnormality applying systematic International Classification of Diseases (ICD) coding
Assess cardiac auscultation repeatedly
Growth and development
Assess growth and development of child to 1 year (optional)
Use standard referenced developmental assessment (optional)
Mortality
Report both overall mortality and malaria-related mortality
Report maternal death
Define perinatal mortality, early neonatal mortality and neonatal mortality. e.g. WHO definition

## Conclusions

Much is still not known about the impact of anti-malarial treatment on the consequences of malaria infection in pregnancy. Every effort should be made to make the most of each treatment episode in an efficacy study by gathering a minimal set of important safety data to fill this knowledge gap. This valuable data can then be pooled with data from other sources of clinical safety information such as pharmacovigilance studies or exposure registry to strengthen safety conclusions. Standardization of assessment and reporting will be a foundation for research with more comparable and reliable outputs and will generate the needed evidence to guide policy.

## Additional files

**Additional file 1.** Search terms for literature review.

**Additional file 2.** PRISMA flowchart.

**Additional file 3.** Summary of the study design and reported outcomes.

**Additional file 4.** Summary of the assessment of non-obstetric acute safety.

**Additional file 5.** Definition and reporting of fetal losses and prematurity of newborns.

**Additional file 6.** Anthropometric and developmental assessment of the newborns.

**Additional file 7.** Assessment and reporting of risk factors of adverse pregnancy outcomes.

## Abbreviations

ABT: artemisinin-based treatments
BMI: body mass index
CDISC: Clinical Data Interchange Standards Consortium
ECG: electrocardiogram
ICD: International Classification of Diseases
LBW: low birth weight
PK: pharmacokinetic
PTB: preterm birth
QBT: quinine-based treatments
RCT: randomized control trial
SGA: small for gestational age
WHO: World Health Organization
