# Supplementary material for: Methodology of assessment and reporting of safety in anti-malarial treatment efficacy studies of uncomplicated falciparum malaria in pregnancy: a systematic literature review
Source: Malar J. 2017 Dec 18;16:491. doi: 10.1186/s12936-017-2136-x (PMC5735519; doi:10.1186/s12936-017-2136-x)
Supplement: Supplementary file 3 — Additional file 3. Summary of the study design and reported outcomes. [file 12936_2017_2136_MOESM3_ESM.pdf]

Additional file 3 Summary of the study design and reported outcomes.

Study<br>Country<br>Study year	Study design<br>Treatment: N	Trimester	Estimation of gestational age	Parity or gravidity reported	Follow up until delivery	Length of follow-up after delivery	ECG	Blood test	Pregnancy outcomes reported
Naing, 1988<br>Myanmar<br>1985-1986<br>[26]	RCT<br>Q: 23<br>AQ: 19	2,3	Not specified	Parity	Weekly	Not followed	Not reported	Routine blood test was carried out, but the results were not reported.	Stillbirth/miscarriage<br>Preterm birth<br>Neonatal mortality
Harinasuta, 1990[a]<br>Thailand<br>INA<br>[27]	RCT<br>Q: 82<br>MFQ: 85	1,2,3	Not specified	INA	Yes	6 months	INA	Blood glucose	Stillbirth/miscarriage<br>Preterm birth<br>Low birth weight (%)<br>Congenital abnormality<br>Child development (6 months)
Nosten, 1993<br>Thailand<br>1989-1990<br>[28]	RCT<br>Q5: 16<br>Q5 + spiramycin: 16	2,3	Not specified	Parity	Not followed	Not followed	Not reported	WBC count at baseline	Stillbirth/miscarriage during the treatment
Sowunmi, 1998<br>Nigeria<br>1994-1997<br>[29]	RCT<br>AM: 23<br>AMMQ: 22	2,3	Ultrasound	Parity	3-6 weekly	3 years	Not reported	Total WBC count, platelet count, ALT, AST, bilirubin, sodium, potassium, chloride, bicarbonate, uric acid, creatinine and blood glucose at baseline and on day 7	Stillbirth/miscarriage<br>Preterm birth<br>Congenital abnormality<br>Birth weight<br>Child development
Bounyasong, 2001<br>Thailand<br>1995-1998<br>[30]	RCT<br>ASMQ: 29<br>Q: 28	2,3	Fundal height + ultrasound	Parity	Weekly	2 years	Not reported	CBC at baseline and daily during the study period, but not reported. Blood glucose.	Calcification of placenta<br>Intra-uterine growth restriction<br>Estimated gestational age at delivery<br>Birth weight<br>Congenital abnormality<br>Child development (up to 2 years)

Additional file 3 continued.

Study<br>Country<br>Study year	Study design<br>Treatment: N	Trimester	Estimation of gestational age	Parity or gravidity reported	Follow up until delivery	Length of follow-up after delivery	ECG	Blood test	Pregnancy outcomes reported
McGready, 2000<br>Thailand<br>1995-1997<br>[31]	RCT<br>Q: 42<br>ASMQ: 66	2,3	Fundal height + Dubowitz	Parity	Weekly	1 year	Not reported	Not reported	Stillbirth/miscarriage<br>Estimated gestational age at delivery<br>Birth weight / low birth weight (%)<br>Placental weight<br>Congenital abnormality<br>Child development<br>Neonatal mortality / Infantile mortality<br>Maternal mortality
McGready, 2001a<br>Thailand<br>1997-2000<br>[32]	RCT<br>QC: 65<br>AS: 64	2,3	Fundal height + Dubowitz	Parity	Weekly	1 year	Not reported	Not reported	Stillbirth/miscarriage<br>Estimated gestational age at delivery<br>Birth weight / low birth weight (%)<br>Congenital abnormality<br>Placental weight<br>Neonatal mortality / infantile mortality<br>Child development
McGready, 2005<br>Thailand<br>2001-2003<br>[33]	RCT<br>AAP: 39<br>Q: 42	2,3	Ultrasound, Dubowitz or Fundal height	Gravidity	Weekly	1 year	Not reported	CBC at baseline	Stillbirth/miscarriage<br>Estimated gestational age at delivery<br>Birth weight / low birth weight (%)<br>Height, head circumference, arm circumference<br>Congenital abnormality<br>Infantile mortality<br>Child development<br>Maternal mortality
Adam, 2004a<br>Sudan<br>2002-2003<br>[34]	RCT<br>Q (low dose): 18<br>Q (standard dose): 24	2,3	Not specified	Parity	Yes	Not followed	Not reported	Blood glucose	Preterm birth<br>Perinatal mortality

Additional file 3 continued.

Study<br>Country<br>Study year	Study design<br>Treatment: N	Trimester	Estimation of gestational age	Parity or gravidity reported	Follow up until delivery	Length of follow-up after delivery	ECG	Blood test	Pregnancy outcomes reported
Kalilani, 2007<br>Malawi<br>2003-2004<br>[35]	RCT<br>ASSP: 47<br>SP: 47<br>SP + azithromycin: 47	2	Ballard score	Gravidity	Yes	Not followed	Not reported	Not reported	Anaemia at delivery<br>Stillbirth/miscarriage<br>Preterm birth<br>Birth weight and low birth weight<br>Congenital abnormality<br>Neonatal death
McGready, 2008<br>Thailand<br>2004-2006<br>[36]	RCT<br>AL: 125<br>AS: 128	2,3	Ultrasound or Dubowitz	Both	Weekly	1 year	Yes (subset)	CBC and biochemistry at baseline and on day 14 (subset of patients)	Stillbirth/miscarriage<br>Estimated gestational age at delivery<br>Birth weight / low birth weight (%)<br>Height, head circumference, arm circumference<br>Congenital abnormality<br>Neonatal neurological test<br>Neonatal / Infantile mortality<br>Child development<br>Maternal mortality
Mutabingwa, 2009<br>Tanzania<br>2004-2006<br>[37]	RCT<br>ASAQ: 83<br>AQSP: 80<br>CD: 81<br>SP: 28	2,3	Dubowitz	Parity	At delivery and 42d after delivery	6 weeks	Yes	Total WBC count with differential, platelet count, creatinine, total bilirubin, ALT and albumin at baseline, on day 3 and 7	Stillbirth/miscarriage<br>Preterm birth<br>Birth weight<br>Congenital abnormality<br>Placenta weight<br>Maternal mortality
Kaye, 2008<br>Uganda<br>2006<br>[38]	RCT<br>AL: 57<br>CD: 57	2,3	Not specified	Gravidity	Not followed	Not followed	Not reported	Not reported	None

Additional file 3 continued.

Study<br>Country<br>Study year	Study design<br>Treatment: N	Trimester	Estimation of gestational age	Parity or gravidity reported	Follow up until delivery	Length of follow-up after delivery	ECG	Blood test	Pregnancy outcomes reported
Piola, 2010<br>Uganda<br>2006-2009<br>[39]	RCT<br>AL: 152<br>Q: 152	2,3	Ultrasound or LMP	Gravidity	Weekly	1 year	Yes	Creatinine, ALT and bilirubin at baseline, on day 7, 14 and 42. CBC at baseline, on day 14 and 42	Stillbirth/miscarriage<br>Preterm birth / gestational age at delivery<br>Birth weight / low birth weight (%)<br>Congenital abnormality<br>Early neonatal mortality<br>Maternal mortality
Carmona-Fonseca, 2013<br>Colombia<br>2008-2011<br>[40]	RCT<br>AL: 15<br>ASMQ: 15	2,3	Not specified	Gravidity	Not followed	Not followed	Not reported	Creatinine, AST, ALT, direct and indirect bilirubin, and platelet 24 hours after the treatment	Maternal mortality
D'Alessandro, 2016<br>Burkina Faso, Ghana, Malawi and Zambia<br>2010-2013<br>[41, 42]	RCT<br>AL: 880<br>ASAQ: 842<br>ASMQ: 848<br>DP: 853	2,3	Fundal height + New Ballard score	Gravidity	Monthly	1 year	Not reported	Total WBC count with differential at baseline, on day 7, 14, 28 and 63. Total bilirubin, ALT and creatinine at baseline, on day 7 and 14.	Stillbirth/miscarriage<br>Preterm birth<br>Birth weight<br>Congenital abnormality<br>Maternal mortality
Osarfo, 2017<br>Ghana<br>2011-2012<br>[43]	RCT<br>ASAQ: 205<br>DP: 212	2,3	ultrasound (or Ballard if not done)	Parity	Yes	6 weeks	Not reported	CBC at baseline, on day 14, 28 and 42	Stillbirth/miscarriage<br>Preterm birth<br>Birth weight / low birth weight<br>Congenital abnormality<br>Neonatal mortality and morbidity<br>Maternal mortality

Additional file 3 continued.

Study<br>Country<br>Study year	Study design<br>Treatment: N	Trimester	Estimation of gestational age	Parity or gravidity reported	Follow up until delivery	Length of follow-up after delivery	ECG	Blood test	Pregnancy outcomes reported
Onyamboko, 2015[a]<br>DRC<br>2013-2014<br>[44]	RCT<br>AL: 48<br>AL5: 48	2,3	Ultrasound	INA	Yes	1 year	INA	Biochemistry	Stillbirth/miscarriage<br>Congenital abnormality<br>Neonatal mortality<br>Child development
Ukah, 2015<br>Nigeria<br>2013<br>[45]	RCT<br>AL: 75<br>ASAQ: 75	2,3	Not specified	Parity	Telephone confirmation after birth	Not followed	Not reported	Not reported	Stillbirth/miscarriage
Iribhogbe, 2017a<br>Nigeria<br>INA<br>[46]	RCT<br>AL: 40<br>ASAQ: 40	2,3	Not specified	Both	No	No	Not reported	Differential WBC count, total and conjugated bilirubin, ALT, AST, ALP, cholesterol) on day 4.	None
CTRI/2009/091/001055[b]<br>India<br>2010-2015<br>[47]	RCT<br>ASMQ (500)<br>ASSP (500)	2,3	INA	INA	Yes	42 days	INA	Biochemistry	Birth outcomes<br>Congenital abnormality
NCT01054248[b]<br>Thailand<br>2010-2016<br>[48]	RCT<br>AL4 (335)<br>ASMQ (335)<br>DP (335)	2,3	Ultrasound (Dubowitz, LMP or fundal height)	Both	Yes	1 year	INA	Full biochemistry and CBC at baseline, on day 0, 14 and 28 (subset of the patients)	Stillbirth/miscarriage<br>Preterm birth<br>Congenital abnormality<br>Birth weight / low birth weight (%)<br>Neonatal and infant mortality<br>Child development
McGready, 2003a<br>Thailand<br>2000-2001<br>[49]	PK<br>AAP: 24	2,3	Fundal height + Dubowitz	Both	Weekly	Not followed	Yes	CBC and blood glucose at baseline. Biochemistry at baseline and 72 h after treatment	Stillbirth/miscarriage<br>Estimated gestational age at delivery / preterm birth<br>Birth weight / low birth weight (%)<br>Congenital abnormality<br>Maternal mortality

Additional file 3 continued.

Study<br>Country<br>Study year	Study design<br>Treatment: N	Trimester	Estimation of gestational age	Parity or gravidity reported	Follow up until delivery	Length of follow-up after delivery	ECG	Blood test	Pregnancy outcomes reported
Adam, 2012<br>Sudan<br>2007-2008<br>[50]	PK study<br>DP: 12<br>(control: 12 matched non-pregnant women)	2,3	LMP+ ultrasound	Parity	Weekly	1 year	Not reported	Urea, AST and ALT at baseline and on day 14	Stillbirth/miscarriage<br>Preterm birth<br>Birth weight<br>Congenital abnormality<br>Child development (up to 1 year)
Onyamboko, 2011<br>DRC<br>2007-2008<br>[51]	PK<br>ASSP: 26<br>(control: 25 non-pregnant women)	2,3	Ultrasound	Parity	Yes	1 year	Not reported	Creatinine, albumin, ALT, AST and alpha-1-acid glycoprotein at baseline and after the first dose of artesunate	Stillbirth/miscarriage<br>Birth weight / low birth weight (%)<br>Congenital abnormality<br>Child development (1 year)
McGready, 2012<br>Thailand<br>2008-2009<br>[52]	PK<br>AS: 20	2,3	Ultrasound	Both	Weekly	1 year	Not reported	CBC and biochemistry at baseline	Stillbirth/miscarriage<br>Estimated gestational age at delivery / preterm birth<br>Birth weight / low birth weight (%)<br>Congenital abnormality<br>Child development
Rijken, 2011<br>Thailand<br>2008<br>[53]	PK<br>DP: 24<br>(control: 24 matched non-pregnant women)	2,3	Ultrasound	Both	Weekly	1 months	Not reported	CBC and biochemistry at baseline and on day 14	Stillbirth/miscarriage<br>Congenital abnormality<br>Neonatal mortality
Valea, 2014<br>Burkina Faso<br>2008-2009<br>[54]	PK<br>ASMQ: 24<br>(control: 24 non-pregnant women)	2,3	LMP or fundal height	Parity	Passive follow-up	Not followed	Not reported	Total WBC count, total bilirubin, ALT, AST, creatinine at baseline and on day 14	Stillbirth/miscarriage<br>Birth weight / low birth weight (%)<br>Congenital abnormality

Additional file 3 continued.

Study<br>Country<br>Study year	Study design<br>Treatment: N	Trimester	Estimation of gestational age	Parity or gravidity reported	Follow up until delivery	Length of follow-up after delivery	ECG	Blood test	Pregnancy outcomes reported
Juma, 2014[a]<br>Kenia<br>2012<br>[55]	PK<br>AL: 45<br>(control: 25 non-pregnant women)	2,3	Not specified	INA	Not followed	Not followed	INA	Not reported	Not planned
Mosha, 2014<br>Tanzania<br>2012<br>[56]	PK<br>AL: 33	2,3	LMP + fundal height	No	Not followed	Not followed	Not reported	WBC count with differential, platelet count, creatinine, ALT and AST at baseline, on day 14 and 28	Stillbirth/miscarriage within 42 days
Nyunt, 2016<br>Uganda<br>2013-2014<br>[57]	PK<br>AL: 30<br>(control: 30 non pregnant adults)	2,3	LMP + ultrasound	No	Not followed	Not followed	Not reported	Not reported	None
Mutagonda, 2017<br>Tanzania<br>2014-2015<br>[58, 59]	PK<br>AL: 60	2,3	Not specified	Gravidity	Not followed	Not followed	Not reported	Urea, creatinine, albumin and bilirubin at baseline	None
Adam, 2004b<br>Sudan<br>2000-2002<br>[60]	Single-arm<br>Q: 26	1	Ultrasound	Parity	Every 2 weeks	1 year	Not reported	Urea, creatinine, albumin and bilirubin at baseline	Stillbirth/miscarriage<br>Preterm birth<br>Birth weight<br>Congenital abnormality<br>Child development (up to 1 year)<br>Infantile mortality

Additional file 3 continued.

Study<br>Country<br>Study year	Study design<br>Treatment: N	Trimester	Estimation of gestational age	Parity or gravidity reported	Follow up until delivery	Length of follow-up after delivery	ECG	Blood test	Pregnancy outcomes reported
Adam, 2004c<br>Sudan<br>1997-2001<br>[61]	Single-arm<br>AM im: 28	1,2,3	Ultrasound	Parity	Every 2 weeks	1 year	Not reported	Urea, creatinine, albumin and bilirubin at baseline	Stillbirth/miscarriage<br>Preterm birth<br>Birth weight<br>Head circumference<br>Congenital abnormality<br>Child development (up to 1 year)<br>Perinatal mortality<br>Maternal mortality
Adegnika, 2005<br>Gabon<br>2003-2004<br>[62]	Single-arm<br>Q: 50	2,3	Not specified	Both	Monthly	Not followed	Not reported	Not reported	None
Adam, 2006<br>Sudan<br>2004-2005<br>[63]	Single-arm<br>ASSP: 32	2,3	Ultrasound	Parity	Every 2 weeks	1 month	Not reported	Not reported	Stillbirth/miscarriage<br>Preterm birth<br>Birth weight<br>Head circumference<br>Congenital abnormality<br>Child development (up to 1 month)<br>Perinatal mortality<br>Maternal mortality
Ndiaye, 2011[a]<br>Senegal<br>2009-2011<br>[64]	Single-arm<br>ASAQ: 28	2,3	Fundal height + Dubowitz	INA	At delivery	9 months	Not reported	WBC count with differential, creatinine, ALT, and bilirubin at baseline, on day 7, 14, 28, 42 and at delivery. Blood glucose at baseline.	Stillbirth/miscarriage<br>Gestational age at delivery<br>Birth weight / low birth weight<br>Height<br>Congenital abnormality<br>Child development (up to 9 months)<br>Perinatal mortality

Additional file 3 continued.

Study<br>Country<br>Study year	Study design<br>Treatment: N	Trimester	Estimation of gestational age	Parity or gravidity reported	Follow up until delivery	Length of follow-up after delivery	ECG	Blood test	Pregnancy outcomes reported
Iribhogbe, 2017b<br>Nigeria<br>INA<br>[65]	Single-arm<br>ASAQ: 42	2,3	Not specified	Both	No	No	Not reported	WBC count with differential, total and conjugated bilirubin, ALT, AST, ALP, cholesterol) on day 4	None
McGready, 1998a<br>Thailand<br>1992-1996<br>[66]	Observational cohort<br>AS: 55<br>AMMQ: 2<br>ASMQ: 23	1,2,3	LMP + fundal height + Dubowitz	No	Weekly	1-2 year	Not reported	Not reported	Stillbirth/miscarriage<br>Gestational age at delivery<br>Congenital abnormality<br>Child development
McGready, 1998b<br>Thailand<br>1991-1996<br>[67]	Observational cohort<br>Q: 204	1,2,3	Dubowitz or LMP	Gravidity	Weekly	Not followed	Not reported	Total WBC count and platelet count at baseline	Stillbirth/miscarriage<br>Gestational age at delivery<br>Birth weight<br>Congenital abnormality
McGready, 2001b<br>Thailand<br>1992-2000<br>[68]	Observational cohort<br>artemisinins: 539	1,2,3	Fundal height + Dubowitz or LMP	Both	Weekly	Not followed	Not reported	Not reported	Stillbirth/miscarriage<br>Estimated gestational age at delivery<br>Birth weight / low birth weight (%)<br>Congenital abnormality<br>Placental weight<br>Maternal mortality

Additional file 3 continued.

Study Country Study year	Study design Treatment: N	Trimester	Estimation of gestational age	Parity or gravidity reported	Follow up until delivery	Length of follow-up after delivery	ECG	Blood test	Pregnancy outcomes reported
Laochan, 2015 Thailand 1994-2009 [69]	collation of an observational cohort and interventional studies‡ AS: 151, AAP: 11, AC: 143, AL: 43, ASMQ: 5, DP 29, Q: 476, QC: 29 MQ: 22	1,2,3	Ultrasound, Dubowitz or Fundal height	Parity	Weekly	Not followed	Not reported	Not reported	None
McGready, 2002 Thailand 1995-2000 [70]	observational Q:204	1	Fundal height + Dubowitz	Both	Weekly	Not followed	Not reported	Not reported	Stillbirth/miscarriage<br>Estimated gestational age at delivery<br>Birth weight / low birth weight (%)<br>Congenital abnormality
McGready, 2003b Thailand 1999-2001 [71]	Observational cohort AAP: 27	1,2,3	Not specified	Both	Weekly	Not followed	Not reported	Not reported	Stillbirth/miscarriage<br>Estimated gestational age at delivery<br>Birth weight / low birth weight (%)<br>Congenital abnormality<br>Neonatal mortality
Villegas, 2005[a] Venezuela 2002-2005 [72]	Observational cohort ASMQ: 27	INA	INA	INA	INA	INA	INA	INA	NA

Additional file 3 continued.

Study<br>Country<br>Study year	Study design<br>Treatment: N	Trimester	Estimation of gestational age	Parity or gravidity reported	Follow up until delivery	Length of follow-up after delivery	ECG	Blood test	Pregnancy outcomes reported
Rijken, 2008<br>Thailand<br>2006-2007<br>[73]	Observational cohort<br>DP: 62	1,2,3	Not specified	Both	Weekly	1 month	Not reported	Not reported	Stillbirth/miscarriage<br>Preterm birth<br>Birth weight / low birth weight (%)<br>Congenital abnormality<br>Neonatal mortality
Rulisa, 2012<br>Rwanda<br>2007-2009<br>[74]	Observational cohort<br>AL: (1072) †	1,2,3	LMP + fundal height + ultrasound(some)	Both	Monthly	Not specified	Not reported	Not reported	Stillbirth/miscarriage<br>Preterm birth<br>Birth weight<br>Length, head circumference<br>Congenital abnormality<br>Neonatal mortality<br>Maternal mortality
Kalilani, 2013<br>Malawi<br>2009-2010<br>[75, 76]	Observational cohort<br>AL: 56	1,2	LMP + fundal height + Ballard score	Gravidity	Monthly	14weeks	Not reported	Not reported	Anaemia at delivery<br>Stillbirth/miscarriage<br>Estimated gestational age at delivery / preterm birth (%)<br>Birth weight / low birth weight (%) / small for gestational age (%)<br>Neonatal death

AAP: artesunate-atovaquone-proguanil, AC: artesunate-clindamycin, AL: artemether-lumefantrine, ALT: alanine aminotransferase, AQ: amodiaquine, AS: artesunate, AST: aspartate aminotransferase, CBC: complete blood count, CD: chlorproguanil-Dapsone, CHQ: chloroquine, DP: dihydroartemisinin(DHA)-piperaquine(PPQ), DRC: Democratic Republic of Congo, ECG: electrocardiography, LMP: last menstrual period, MQ: mefloquine, N: number of pregnant women who completed the follow-up, NA: data not available, PCR: polymerase chain reaction, PK: pharmacokinetic study, Q: quinine, QC: quinine-clindamycin, RCT: randomised controlled trial, SP: sulfadoxine-pyrimethamine. WBC: white blood cell.

† This number includes patients without parasitological confirmation. The exact number of parasitologically confirmed patients is not known. ‡This study included patients enrolled in other studies. [a]: Conference abstract. [b]: Registered clinical trial. Planned number of participants is shown. NCT01054248 included *P. vivax* cases.
