# Supplementary material for: Methodology of assessment and reporting of safety in anti-malarial treatment efficacy studies of uncomplicated falciparum malaria in pregnancy: a systematic literature review
Source: Malar J. 2017 Dec 18;16:491. doi: 10.1186/s12936-017-2136-x (PMC5735519; doi:10.1186/s12936-017-2136-x)
Supplement: Supplementary file 4 — Additional file 4. Summary of the assessment of non-obstetric acute safety. [file 12936_2017_2136_MOESM4_ESM.pdf]

Additional file 4 Summary of the assessment of non-obstetric acute safety.

Vital signs other than body temperature were assessed in 18 studies (38%, 18/48), and only two studies reported the results after treatment [41,54]. Five studies (10%, 5/48) assessed maternal neurological examination given early safety concerns for artemisinin derivatives [30,31,61,63,66], and three studies reported the results [30,61,66].

Thirty-nine studies (81% ,39/48) reported symptoms after treatment. Seven studies (15%, 7/48) did not report symptoms [26,56,58,61,62,69,75] and no information was available from two conference abstracts [44,55].

Biochemistry was assessed in 20 studies (42%, 20/48) and blood glucose was assessed in six studies [27,29,30,34,49,64]. White blood cell count was assessed in 19 studies (40%, 19/48). One study reported having assessed routine blood tests without specification [26]. Urinalysis was assessed in six studies (14%, 6/48) but none of them reported the results [26,37,39,40,61,63]. One study reported urine colour [38].

Electrocardiogram (ECG) was assessed in three RCTs (14%, 3/22) and one PK study (10%, 1/10) [36,37,39,49]. ECG was performed at baseline in all four studies. Three studies assessed one time after drug administration (on day 2 [39], 1 hour after the last dose [36], and on day 3 [37]) One study assessed four times after drug administration: 1 hours after each dose and 24 hours after the last dose [49].
