# Supplementary material for: Methodology of assessment and reporting of safety in anti-malarial treatment efficacy studies of uncomplicated falciparum malaria in pregnancy: a systematic literature review
Source: Malar J. 2017 Dec 18;16:491. doi: 10.1186/s12936-017-2136-x (PMC5735519; doi:10.1186/s12936-017-2136-x)
Supplement: Supplementary file 5 — Additional file 5. Definition and reporting of fetal losses and prematurity of newborns. [file 12936_2017_2136_MOESM5_ESM.pdf]

Additional file 5 Definition and reporting of fetal losses and prematurity of newborns.

Study	Miscarriage		Stillbirth		EGA	Prematurity (preterm)		Perinatal death		Neonatal death	
	Definition	Report	Definition	Report	Report	Definition	Report	Definition	Report	Definition	Report
Naing, 1988 [26]	NR	Yes	NR	NR	NR	NR	Yes	NR	NR	NR	Yes
Harinasuta, 1990 [27]	INA	Yes (0)*	INA	Yes (0)*	NR	INA	Yes	INA	INA	INA	INA
Sowunmi, 1998 [29]	NR	Yes (0)*	NR	Yes (0)*	NR	NR	Yes (0)	NR	NR	NR	NR
Bounyasong, 2001 [30]	NR	Yes (0)*	NR	Yes (0)*	Yes	NR	NR	NR	NR	NR	NR
McGready, 2000 [31]	NR	Yes	NR	Yes (0)	Yes	<37w	NR	NR	NR	NR	Yes‡
McGready, 2001a [32]	NR	Yes (0)	NR	Yes	Yes	<37w	NR	NR	NR	NR	Yes‡
McGready, 2005 [33]	<28w	Yes (0)	≥28w	Yes	Yes	<37w	Yes	NR	NR	NR	Yes‡
Adam, 2004a [34]	NR	NR	NR	NR	NR	<37w	Yes	NR	NR	NR	Yes
Kalilani, 2007 [35]	<28w	Yes (0)	≥28w	Yes	Yes	<37w	NR	Yes‡	Yes	≤27 days of life	Yes
McGready, 2008 [36]	NR	Yes	NR	Yes	Yes	<37w	Yes	NR	NR	0-32 days	Yes‡
Mutabingwa, 2009 [37]	NR	NR	NR	Yes	NR	NR	NR	NR	NR	NR	Yes
Piola, 2010 [39]	<20w (spontaneous abortions) >20w (intrauterine fetal deaths)	Yes	>21w (protocol)	Yes	Yes	Prematurity (<37w) Severe prematurity (<28w)	Yes	NR	NR	early neonatal death (1 week after birth)	Yes
D'Alessandro, 2016 [41]	NR	Yes	NR	Yes	NR	<37w	Yes	NR	NR	NR	NR
Osarfo, 2017 [43]	<28w	Yes	≥28w	Yes	NR	<37w	Yes	NR	NR	NR	Yes
Onyamboko, 2015 [44]	INA	INA	INA	Yes	NR	INA	INA	INA	INA	INA	Yes
Ukah, 2015 [45]	NR	Yes	NR	Yes	NR	NR	NR	NR	NR	NR	NR

Additional file 5 continued

Study	Miscarriage		Stillbirth		EGA	Prematurity (preterm)		Perinatal death		Neonatal death	
	Definition	Report	Definition	Report	Report	Definition	Report	Definition	Report	Definition	Report
CTRI/2009/091/001055 [47]	INA	INA	INA	INA	INA	INA	INA	INA	INA	INA	INA
NCT01054248 [48]	INA	Planned	INA	Planned	INA	INA	Planned	INA	INA	INA	Planned
McGready, 2003a [49]	NR	Yes (0)*	NR	Yes (0)*	Yes	<37w	Yes (0)	NR	NR	NR	NR
Adam, 2012 [50]	<28w	Yes (0)	NR	NR	NR	28-37w	Yes	NR	NR	NR	NR
Onyamboko, 2011 [51]	NR	Yes (0)*	NR	Yes (0)*	NR	NR	NR	NR	NR	NR	NR
McGready, 2012 [52]	NR	Yes (0)*	NR	Yes (0)*	Yes	NR	Yes	NR	NR	NR	Yes (0)
Rijken, 2011 [53]	NR	Yes (0)*	NR	Yes (0)*	NR	NR	NR	NR	NR	1 month	Yes (0)
Valea, 2014 [54]	NR	NR	NR	NR	NR	NR	NR	NR	NR	NR	NR
Adam, 2004b [60]	<28w	Yes	NR	NR	NR	28-37w	NR	from 28 weeks until 1 week after delivery	NR	NR	Yes‡
Adam, 2004c [61]	<28w	Yes (0)	NR	Yes (0)	NR	NR	Yes	NR	NR	NR	Yes
Adegnika, 2005 [62]	NR	NR	NR	NR	NR	NR	NR	NR	NR	NR	NR
Adam, 2006 [63]	NR	Yes (0)	NR	Yes (0)	NR	NR	Yes (0)	NR	NR	NR	Yes
Ndiaye, 2011 [64]	NR	Yes (0)*	NR	Yes (0)*	Yes	NR	Yes	NR	NR	NR	Yes
McGready, 1998a [66]	<28w	Yes	≥28w	Yes	Yes	NR	NR	NR	NR	NR	NR
McGready, 1998b [67]	<28w	Yes	NR	Yes	Yes	NR	NR	NR	NR	NR	Yes (0)
McGready, 2001b [68]	<28w	Yes	≥28w	Yes	Yes	<37w	NR	NR	NR	NR	NR
Laochan, 2015 [69]	NR	NR	NR	NR	NR	NR	NR	NR	NR	NR	NR
McGready, 2002 [70]	<28w	Yes	≥28w	Yes	Yes	<37w	NR	NR	NR	NR	NR
McGready, 2003b [71]	NR	Yes (0)*	NR	Yes (0)*	Yes	NR	Yes	NR	NR	NR	Yes‡
Rijken, 2008 [73]	NR	Yes (0)*	NR	Yes (0)*	NR	<37w	Yes	NR	NR	1 month	Yes
Rulisa, 2012 [74]	NR	Yes	NR	Yes	NR	NR	Yes	NR	NR	≤ 7 days	Yes
Kalilani, 2013 [75]	NR	NR	NR	Yes	Yes	<37w	Yes	NR	NR	NR	Yes

EGA: estimated gestational age at delivery. INA: information not available (conference abstract or registered trial), NR: not reported.
* No fetal losses were reported. ‡ include deaths after a month. ‡Total of abortion, stillbirth and neonatal deaths.
