# Supplementary material for: Methodology of assessment and reporting of safety in anti-malarial treatment efficacy studies of uncomplicated falciparum malaria in pregnancy: a systematic literature review
Source: Malar J. 2017 Dec 18;16:491. doi: 10.1186/s12936-017-2136-x (PMC5735519; doi:10.1186/s12936-017-2136-x)
Supplement: Supplementary file 6 — Additional file 6. Anthropometric and developmental assessment of the newborns. [file 12936_2017_2136_MOESM6_ESM.pdf]

Additional file 6 Anthropometric and developmental assessment of the newborns.

Study	Birthweight	Timing	Adjustment for Sex	Adjustment for Gestational age	Twins	Minimal precision of scale	LBW Definition	LBW Reported	Height	Length of follow-up after delivery	Developmental test
Naing, 1988 [26]	No	NoBW	NoBW	NoBW	NR	NoBW	NR	NR	NR	Not followed	-
Harinasuta, 1990 [27]	Yes	NR	No	No	INA	INA	<2500g	Yes	INA	6 months	Not specified
Sowunmi, 1998 [29]	Yes	NR	No	No	NR	NR	NR	NR	NR	3 years	Yes [71,72]
Bounyasong, 2001 [30]	Yes	24h	No	No	NR	NR	NR	NR	NR	2 years	Denver developmental screening test [73]
McGready, 2000 [31]	Yes	NR	No	No	Body weight and ega were reported in singletons only.	50g	NR	Yes	Assessed, but not reported.	1 year	Basic developmental milestones
McGready, 2001a [32]	Yes	NR	No	No	Body weight were reported in singletons only.	50g	NR	Yes	Assessed, but not reported	1 year	Developmental milestones
McGready, 2005 [33]	Yes	24h	Assessed, but not reported	No	Body weight and ega were reported in singletons only.	NR	<2500g	Yes	Yes	1 year	Yes [74]
Adam, 2004a [34]	No	NoBW	NoBW	NoBW	NR	NoBW	NR	NR	NR	NR	-
Kalilani, 2007 [35]	Yes	24h	No	No	Women with multiple gestations were excluded from the study.	To the nearest gram	<2500g	Yes	NR	Not followed	-
McGready, 2008 [36]	Yes	72h	No	Term low birthweight	Body weight and ega were reported in singletons only.	NR	<2500g	Yes	Yes	1 year	Shoklo developmental score [74]
Mutabingwa, 2009 [37]	Yes	NR	No	No	Women with multiple gestations were excluded from the study.	NR	NR	NR	NR	6 weeks	Not specified
Piola, 2010 [39]	Yes	NR	No	Term low birthweight	Proportion of multiple births was planned to be reported but not.	NR	<2500g	Yes	Assessed, but not reported	1 year	Yes [75]
D'Alessandro, 2016 [41]	Yes	72h	No	No	Analysis of body weight was restricted to singleton births.	Digital baby scale	<2500g	Yes	NR	1 year	Not specified

Additional file 6 continued.

Study	Birthweight	Timing	Adjustment for		Twins	Minimal precision of scale	LBW		Height	Length of follow-up after delivery	Developmental test
			Sex	Gestational age			Definition	Reported			
Osarfo, 2017 [43]	Yes	NR	No	No	Women with multiple gestations were excluded from the study.	NR	<2500g	Yes	Assessed, but not reported	6 weeks	Not specified
Onyamboko, 2015 [44]	INA	INA	INA	INA	Women with multiple gestations were excluded from the study.	INA	INA	INA	INA	1 year	Not specified
Ukah, 2015 [45]	No	NoBW	NoBW	NoBW	NR	NoBW	NR	NR	NR	Not followed	-
CTRI/2009/091/001055 [47]	INA	INA	INA	INA	INA	INA	INA	INA	INA	42 days	Not specified
NCT01054248 [48]	Yes	As soon as possible	Planned	No	INA	INA	INA	Planned	Planned	1 year	Yes [74]
McGready, 2003a [49]	Yes	<5 days	Assessed, but not reported	No	All delivered singleton.	NR	<2500g	Yes	NR	Not followed	-
Adam, 2012 [50]	Yes	NR	Assessed, but not reported	No	NR	NR	<2500g	Yes	NR	1 year	Neurologic developmental assessment
Onyamboko, 2011 [51]	Yes	NR	No	No	All delivered singleton.	NR	≤2500g	Yes	NR	1 year	Not specified
McGready, 2012 [52]	Yes	72h	Assessed, but not reported	No	All delivered singleton.	10g	<2500g	Yes	NR	1 year	Yes [74]
Rijken, 2011 [53]	No	NoBW	NoBW	NoBW	All delivered singleton. Birthweight was not reported.	± 10g	NR	NR	Assessed, but not reported	1 months	Not specified
Valea, 2014 [54]	Yes	NR	No	No	NR	10g	<2500g	NR	NR	Not followed	-
Adam, 2004b [60]	Yes	NR	No	No	NR	NR	NR	NR	NR	1 year	Not specified
Adam, 2004c [61]	Yes	24h	No	No	NR	NR	NR	NR	NR	1 year	Not specified
Adegnika, 2005 [62]	No	NoBW	NoBW	NoBW	NR	NoBW	NR	NR	NR	Not followed	-
Adam, 2006 [63]	Yes	24h	No	No	NR	NR	NR	NR	NR	1 month	Not specified

Additional file 6 continued.

Study	Birthweight	Timing	Adjustment for Sex	Adjustment for Gestational age	Twins	Minimal precision of scale	LBW Definition	LBW Reported	Height	Length of follow-up after delivery	Developmental test
Ndiaye, 2011 [64]	Yes	48h	No	No	NR	NR	NR	NR	Yes	9 months	Not specified
McGready, 1998a [66]	Yes, in methods but no results	NoBW	NoBW	NoBW	Twins were included. Birthweight was not reported.	NoBW	<2500g	NR	NR	1-2 year	Not specified
McGready, 1998b [67]	Yes	NR	No	No	NR	NR	NR	NR	NR	Not followed	-
McGready, 2001b [68]	Yes	NR	No	No	Body weight and ega were reported in singletons only.	NR	<2500g	Yes	NR	Not followed	-
Laochan, 2015 [69]	No	NoBW	NoBW	NoBW	NR	NoBW	NR	NR	NR	Not followed	-
McGready, 2002 [70]	Yes	NR	No	No	Body weight and ega were reported in singletons only.	NR	<2500g	Yes	NR	Not followed	-
McGready, 2003b [71]	Yes	NR	No	No	All delivered singleton.	NR	<2500g	Yes	NR	Not followed	-
Rijken, 2008 [73]	Yes	3d	No	No	Body weight was reported in singletons only.	NR	<2500g	Yes	NR	1 month	Not specified
Rulisa, 2012 [74]	Yes	NR	No	Term low birthweight	Number of twins was reported, but it is unclear whether they were included in birthweight.	NR	NR	NR	Yes	Not specified	WHO motor development milestones [76]
Kalilani, 2013 [75]	Yes	NR	Yes*	Weight for gestational age*	Placental malaria was reported in singletons only. It is unclear for other assessment (e.g. body weight).	NR	<2500g	Yes	NR	14weeks	Not specified

ega: estimated gestational age. INA: information not available (conference abstract or registered clinical trial). LBW: low birth weight. NoBW: birth weight was not reported. NR: Not reported (information was not reported in the publication). -: not applicable because newborns were not followed. *: WHO standard growth curve was used.
