# Supplementary material for: Methodology of assessment and reporting of safety in anti-malarial treatment efficacy studies of uncomplicated falciparum malaria in pregnancy: a systematic literature review
Source: Malar J. 2017 Dec 18;16:491. doi: 10.1186/s12936-017-2136-x (PMC5735519; doi:10.1186/s12936-017-2136-x)
Supplement: Supplementary file 7 — Additional file 7. Assessment and reporting of risk factors of adverse pregnancy outcomes. [file 12936_2017_2136_MOESM7_ESM.pdf]

Additional file 7 Assessment and reporting of risk factors of adverse pregnancy outcomes.

Study	Smoking	Marital status	Educational level	Height	Nutrition status	Chronic diseases
Naing, 1988 [26]	NR	NR	NR	NR	NR	NR
Harinasuta, 1990 [27]	INA	INA	INA	INA	INA	INA
Nosten, 1993 [28]	NR‡	NR‡	NR‡	NR‡	NR‡	NR‡
Sowunmi, 1998 [29]	NR	NR	NR	NR	NR	NR
Bounyasong, 2001 [30]	NR	NR	NR	Yes	NR	NR
McGready, 2000 [31]	NR	NR	NR	NR	NR	Women with a history of mental disorder excluded.
McGready, 2001a [32]	NR	NR	NR	NR	NR	Women with major liver or kidney disease were excluded.
McGready, 2005 [33]	NR	NR	NR	NR	NR	Women with chronic diseases were excluded from the study.
Adam, 2004a [34]	NR	NR	NR	NR	NR	NR
Kalilani, 2007 [35]	NR	Yes	Yes	NR	NR	Women with a history of chronic disease or pregnancy complications were excluded from the study. HIV status was assessed.
McGready, 2008 [36]	Yes	NR	NR	NR	BMI	Women with chronic diseases were excluded from the study.
Mutabingwa, 2009 [37]	NR	NR	Yes	NR	NR	HIV status was assessed. Women with concomitant disease masking assessment of the response to treatment were excluded.
Kaye, 2008 [38]	NR‡	NR‡	Yes	NR‡	NR‡	NR‡
Piola, 2010 [39]	Recorded but NR.	NR	NR	NR	NR	HIV patient was enrolled.
Carmona-Fonseca, 2013 [40]	NR‡	NR‡	NR‡	NR‡	NR‡	NR‡
D'Alessandro, 2016 [41, 42]	NR	NR	NR	NR	NR	Women with chronic diseases were excluded. HIV positive patients were enrolled only if she received neither antiretroviral treatment nor cotrimoxazole.
Osarfo, 2017 [43]	NR	NR	Yes	Yes, but not reported	NR	Women with chronic diseases (diabetes, hypertension, renal/cardiac) or HIV were excluded from the study.
Onyamboko, 2015 [44]	INA	INA	INA	INA	INA	HIV positive women and women with medical conditions requiring concomitant drug treatment were excluded from the study.
Ukah, 2015 [45]	NR	NR	NR	NR	NR	HIV was assessed and reported.
Iribhogbe, 2017a [46]	NR‡	NR‡	NR‡	Yes	BMI	Women with known severe underlying diseases were excluded from the study.‡
CTRI/2009/091/001055 [47]	INA	INA	INA	INA	INA	Women with known chronic diseases were excluded from the study.
NCT01054248 [48]	Yes	INA	Yes (literacy)	Yes	INA	Women with a history or known liver diseases or other chronic diseases were excluded.
McGready, 2003a [49]	NR	NR	NR	NR	NR	Women with chronic diseases were excluded from the study.
Adam, 2012 [50]	NR	NR	NR	Yes	NR	Women with chronic diseases were excluded.
Onyamboko, 2011 [51]	NR	NR	Yes	Yes	BMI	Women with chronic diseases (diabetes, hypertension, etc.) or HIV were excluded.

Additional file 7 continued.

Study	Smoking	Marital status	Educational level	Height	Nutrition status	Chronic diseases
McGready, 2012 [52]	Yes	NR	NR	NR	BMI	NR
Rijken, 2011 [53]	Yes	NR	NR	Yes	NR	NR
Valea, 2014 [54]	NR	Yes	Yes	Yes	BMI	Patients with chronic medical conditions requiring special care beyond what the study could provide were excluded.
Juma, 2014 [55]	INA	INA	INA	INA	INA	Patients with a history of heart disease or arrhythmia were excluded.
Mosha, 2014 [56]	No history of smoking.	NR‡	NR‡	Yes	BMI	No history of chronic disease.
Nyunt, 2016 [57]	NR‡	NR‡	NR‡	NR	BMI	Women with significant other illness including HIV were excluded from the study. ‡
Mutagonda, 2016 [58, 59]	NR‡	NR‡	NR‡	NR‡	NR‡	NR‡
Adam, 2004b [60]	NR	NR	NR	NR	NR	NR
Adam, 2004c [61]	NR	NR	NR	NR	NR	NR
Adegnika, 2005 [62]	NR	NR	NR	NR	NR	NR
Adam, 2006 [63]	NR	NR	NR	NR	NR	NR
Ndiaye, 2011 [64]	NR	NR	NR	NR	NR	Patients with underlying hepatic or renal illness were excluded.
Iribhogbe, 2017b [65]	NR‡	NR‡	NR‡	Yes	BMI	Women with known severe underlying diseases were excluded from the study. ‡
McGready, 1998a [66]	NR	NR	NR	NR	NR	NR
McGready, 1998b [67]	NR	NR	NR	Yes	NR	NR
McGready, 2001b [68]	NR	NR	NR	NR	NR	NR
Laochan, 2015 [69]	NR‡	NR‡	NR‡	NR‡	NR‡	NR‡
McGready, 2002 [70]	NR	NR	NR	NR	NR	NR
McGready, 2003b [71]	NR	NR	NR	NR	NR	NR
Villegas, 2005 [72]	INA	INA	INA	INA	INA	INA
Rijken, 2008 [73]	NR	NR	NR	NR	NR	NR
Rulisa, 2012 [74]	NR	NR	NR	NR	NR	Diabetes was reported
Kalilani, 2013 [75, 76]	NR	Yes	Yes	NR	NR	Patients with major illness and HIV-positive patients receiving cotrimoxazole were excluded from the study.

INA: information not available (conference abstract or registered clinical trial). NR: not reported. ‡: pregnancy outcomes were not reported.
